# Enhanced UV-C Detection Performance of Perovskite Photodetectors via Controlled Surface Patterning

**DOI:** 10.3390/ma19194234

**Published:** 2026-10-06

**Authors:** Jun Seo Kim, Sangmo Kim, Hyung Wook Choi

**Affiliations:** 1 Department of Electrical Engineering, Gachon University, Seongnam 13120, Republic of Korea; skswnstj0916@naver.com; 2 Research and Development, One Tech. Solution Incorporation, Gunsan 54001, Republic of Korea

**Keywords:** patterning, perovskites, photodetectors, ultraviolet, UV-C, surface engineering

## Abstract

**Highlights:**

**What are the main findings?**

- Perovskite patterning selectively thins the surface while maintaining the main crystalline perovskite phase.
- Among the investigated conditions, a 7 min patterning time with 0.5 mm patterns provided the highest mean device performance.
- Patterning reduces dark current while enhancing photocurrent and key performance metrics.

**What are the implications of the main findings?**

- Controlled UV-C can serve as a surface-engineering tool rather than only a degradation source.
- Surface thinning may modify the optical and carrier-transport pathways associated with the observed photoresponse.
- Mask-assisted UV patterning provides a simple route for perovskite optoelectronic optimization.

**Abstract:**

Ultraviolet (UV)-C photodetectors based on perovskite semiconductors are attractive because of their solution processability and excellent optoelectronic properties, but UV-induced degradation limits conventional UV-based processing. Here, controlled ultraviolet light exposure is repurposed as a surface-engineering and patterning strategy. Shadow-mask-assisted irradiation selectively thinned the perovskite surface, while X-ray diffraction and UV–vis spectroscopy indicated that the main crystalline perovskite phase and overall optical absorption characteristics remained largely unchanged. Patterning conditions were systematically varied using patterning times of 3–10 min and circular pattern diameters of 0.3–1.0 mm. The optimized condition of a 7 min patterning time and a 0.5 mm pattern diameter reduced the dark current and enhanced the photocurrent, resulting in improved responsivity, external quantum efficiency, and specific detectivity. Cross-sectional microscopy and profilometry confirmed controllable surface thinning without macroscopic delamination, while XRD indicated preservation of the detectable crystalline perovskite phase under the investigated conditions. The observed performance enhancement is discussed in terms of possible changes in near-surface electrical losses and optical/carrier-transport pathways associated with controlled surface thinning. These results demonstrate that controlled ultraviolet light irradiation can function as a surface-modification strategy for improving perovskite UV photodetectors while maintaining the principal crystallographic and optical characteristics under the investigated conditions.

## 1. Introduction

Perovskite semiconductors have emerged as promising materials for ultraviolet (UV) photodetection, owing to their high absorption coefficients, tunable bandgaps, and low-temperature solution processability [1,2,3]. Recent advances in perovskite-based optoelectronic devices have further demonstrated the versatility of perovskite materials, particularly through their tunable optical properties and compatibility with diverse device architectures [4]. Conventional UV photodetectors have primarily relied on inorganic wide-bandgap semiconductors, such as Ga_2_O_3_, GaN, and ZnO, which offer strong UV absorption and intrinsic solar-blind characteristics. However, these materials typically require high-temperature processing and complex fabrication procedures, which limit their scalability and compatibility with flexible or large-area substrates [5,6,7]. By contrast, perovskite-based photodetectors provide simplified solution-based fabrication and versatile material tunability, making them attractive alternatives for next-generation UV-sensing applications. Despite these advantages, the environmental and photoinduced instability of perovskites has remained a critical challenge, leading to the widespread perception that UV exposure should be avoided during both operation and processing [8,9,10,11]. However, this inherent UV instability fundamentally limits the use of UV-based processing techniques, such as photolithography, in perovskite devices.

In particular, high-energy UV irradiation can accelerate degradation in perovskite films, which presents a challenge for their use in deep-UV sensing environments [12,13,14,15,16].

Surface and interfacial defects in perovskite films can act as recombination centers and contribute to trap-assisted and nonradiative losses [17,18,19,20].

Conventional patterning techniques, including photolithography, laser-assisted patterning, and nanoimprinting, have been investigated for defining micro- and nanoscale features in perovskite materials [21,22,23]. More recently, photoinduced approaches have also been explored for spatially controlled modification and patterning of perovskite materials, demonstrating that light exposure can be utilized as an active processing tool when carefully controlled [24]. However, their application remains challenging because perovskites are sensitive to irradiation, solvents, and other processing conditions that can induce structural or chemical degradation.

Consequently, most strategies for improving UV photodetector performance have focused on material stabilization or encapsulation to suppress degradation, whereas patterning approaches have remained largely unexplored in perovskite-based UV photodetectors.

In this work, we employ a UV-light-assisted patterning technique as a fundamentally different strategy to selectively modify the surface region of mixed-cation perovskite films. By carefully tuning the patterning conditions, the main crystalline perovskite phase was maintained while the device performance was enhanced. These findings provide new insights into the controlled use of UV irradiation for optical pathway engineering in perovskite-based UV photodetectors.

## 2. Materials and Methods

### 2.1. Substrate Preparation

The ITO-coated quartz substrates (TMA CO., LTD, Goyang-si, Republic of Korea) were sequentially cleaned via ultrasonication in a detergent solution, deionized (DI) water, ethanol (≥99.5%; Sigma-Aldrich, St. Louis, MO, USA), and isopropyl alcohol (IPA). The cleaned substrates were dried at 80 °C for 1 h and subsequently subjected to UV–ozone surface treatment for 15 min to improve the surface wettability prior to film deposition.

### 2.2. Deposition of Electron-Transport Layer

A SnO_2_ electron-transport layer was deposited onto the cleaned ITO substrates by spin-coating a diluted SnO_2_ colloidal solution (15 wt% in water; Alfa Aesar, Seoul, Republic of Korea) containing sodium dodecylbenzenesulfonate (SDBS; Sigma-Aldrich, St. Louis, MO, USA) as an additive. The SnO_2_ solution was prepared by diluting 1.2 mL of a 15 wt% SnO_2_ colloidal dispersion with 5.2 mL of DI water, followed by the addition of 1 mg of SDBS. The SnO_2_ layer was spin-coated at 2000 rpm for 25 s and annealed at 150 °C for 30 min. After annealing, the substrates were treated with UV–ozone for 15 min. All SnO_2_ processing conditions were identical to those used in our previous work.

### 2.3. Preparation and Deposition of Mixed-Cation Perovskite Active Layer

**Figure 1:**
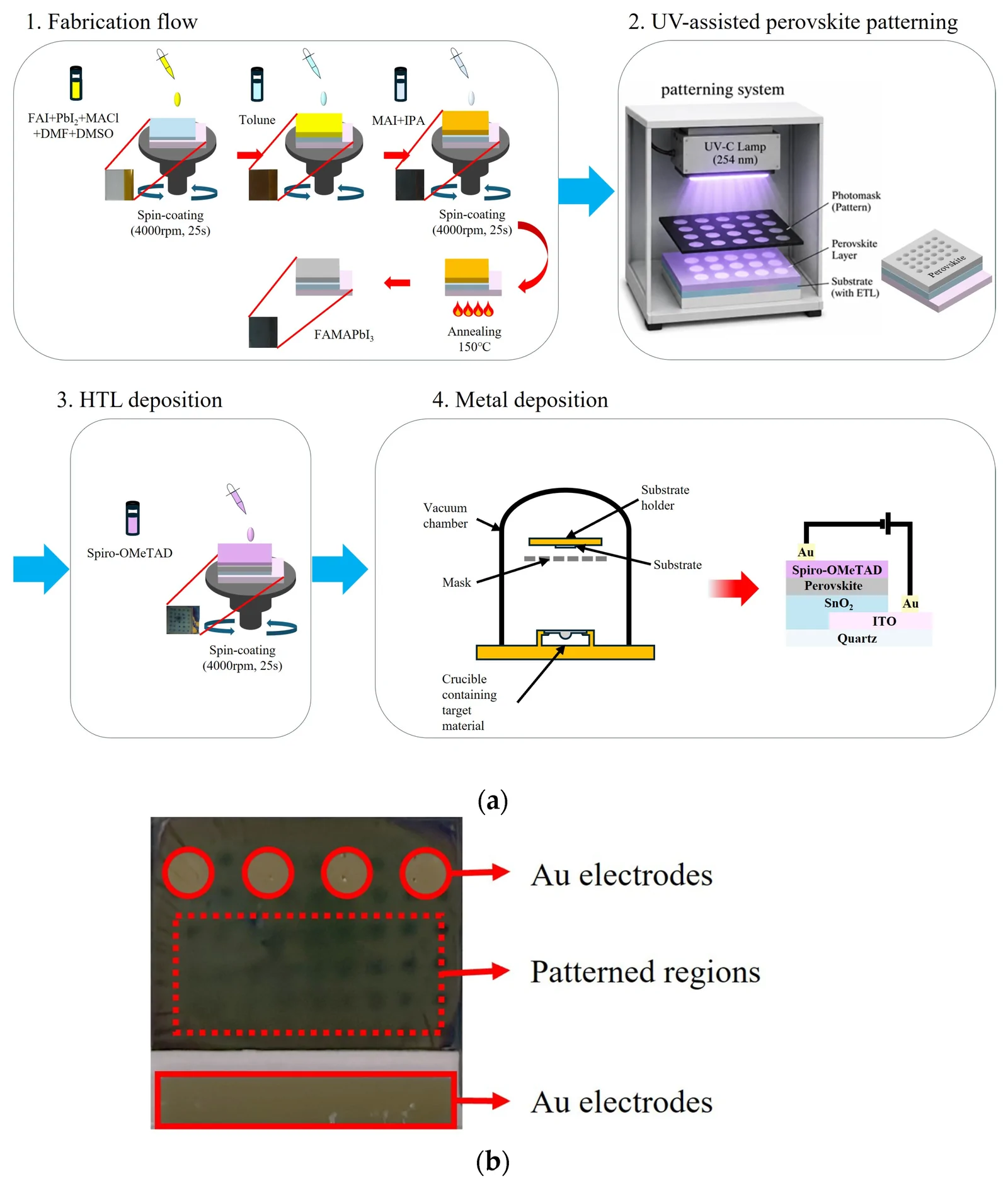
Device architecture and fabrication process of patterned perovskite photodetectors. (**a**) Schematic of the device architecture and fabrication process, including perovskite film formation, patterning, hole-transport layer deposition, and Au top-electrode deposition; (**b**) top-view photograph of the completed patterned perovskite photodetector, showing the Au top electrodes and patterned region in the final device.

The overall device architecture and fabrication process are illustrated in Figure 1. The schematic illustration of the UV-C patterning system in the UV-assisted perovskite patterning panel of Figure 1 was generated with the assistance of ChatGPT (GPT-5.6 Sol, OpenAI) and subsequently reviewed and edited by the authors. A precursor solution was prepared by dissolving lead iodide (PbI_2_; Sigma-Aldrich, St. Louis, MO, USA) and formamidinium iodide (FAI; Sigma-Aldrich, St. Louis, MO, USA) in a solvent mixture of dimethyl sulfoxide (DMSO; Sigma-Aldrich, St. Louis, MO, USA) and N,N-dimethylformamide (DMF; Sigma-Aldrich, St. Louis, MO, USA) at a volume ratio of 10:1. Methylammonium chloride (MACl; GreatCell Solar, Queanbeyan, Australia) was used as an additive to promote film crystallization.

The precursor solution was spin-coated onto the SnO_2_-coated substrates at 4000 rpm for 25 s. Subsequently, a methylammonium iodide (MAI; Sigma-Aldrich, St. Louis, MO, USA) solution in isopropyl alcohol (IPA; Sigma-Aldrich, St. Louis, MO, USA) was spin-coated under the same conditions to induce a two-step conversion process. The resulting films were thermally annealed at 150 °C for 15 min to form mixed-cation perovskite (FAMA) films.

After annealing, patterning was performed under controlled patterning conditions using a shadow mask.

### 2.4. UV-Light-Assisted Patterning of Perovskite Films

**Figure 2:**
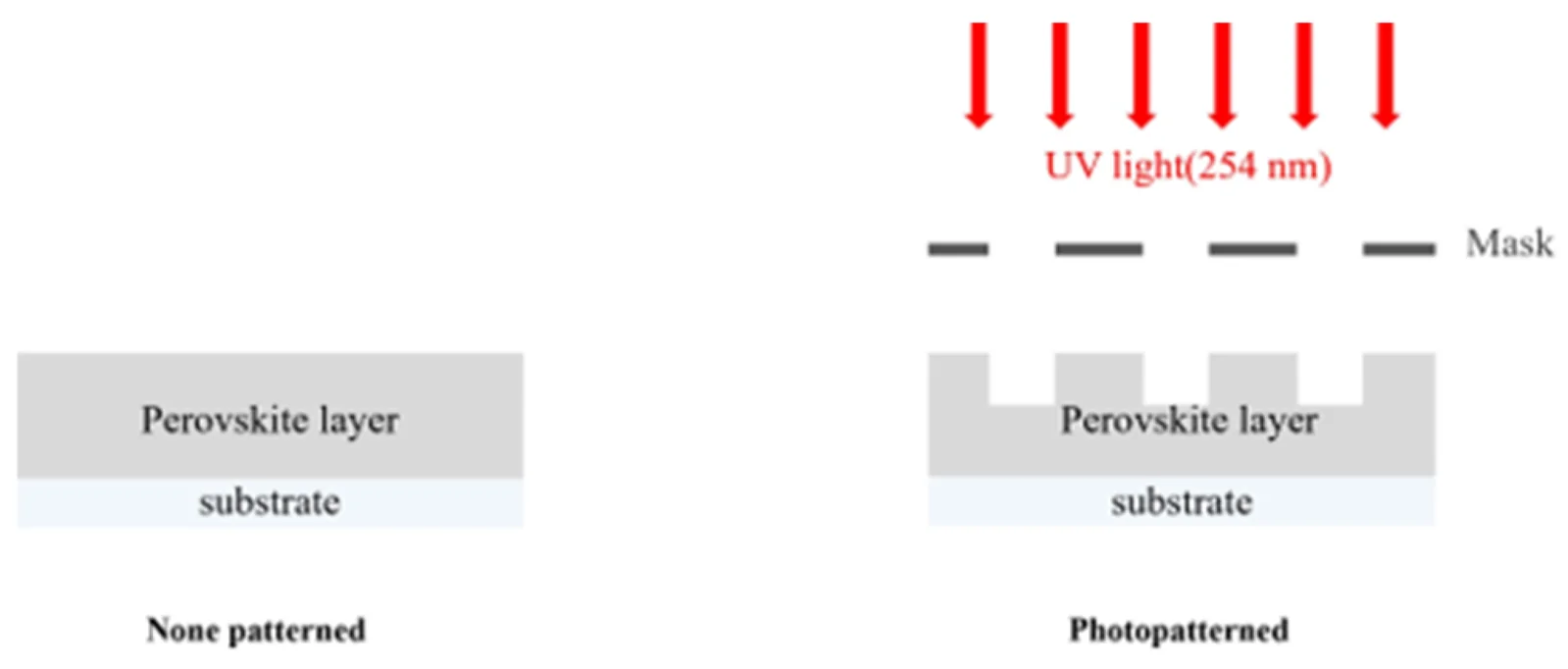
Schematic illustration of the UV-light-assisted patterning system. UV irradiation was applied through a shadow mask to produce a patterned perovskite layer with well-defined circular regions.

The UV-light-assisted patterning system is schematically illustrated in Figure 2. Patterning was carried out using a UV-light-assisted patterning system designed to deliver controlled UV irradiation through a mask.

Patterning was carried out using a UV-light-assisted patterning system designed to deliver controlled UV irradiation through a mask. Ultraviolet light irradiation was performed using a UV-C lamp (18 W, λ = 254 nm, LSE Lighting) positioned approximately 15 cm from the perovskite surface. A mask containing circular patterns with diameters of 0.3, 0.5, and 1.0 mm was placed in direct contact with the perovskite layer to define the patterned regions. The UV patterning time was systematically varied from 3 to 10 min (3, 7, and 10 min) to optimize the patterning conditions, and the irradiation environment was kept constant to ensure reproducibility.

After optimization of the pattern diameter, all subsequent patterned devices were fabricated using 63 circular patterns with a diameter of 0.5 mm.

The perovskite layer was patterned by ultraviolet light irradiation before deposition of the Spiro-OMeTAD hole-transport layer and Au top electrodes. Therefore, the patterned regions were defined directly on the perovskite surface and were not formed on or through the Au electrodes.

### 2.5. Hole-Transport Layer and Top-Electrode Deposition

A hole-transport layer was deposited onto the perovskite films by spin-coating a spiro-OMeTAD (Sigma-Aldrich, St. Louis, MO, USA) solution at 2000 rpm for 25 s. The spiro-OMeTAD solution was prepared by dissolving 72.3 mg of spiro-OMeTAD in 1 mL of chlorobenzene (Sigma-Aldrich, St. Louis, MO, USA), followed by the addition of 28.8 μL of 4-tert-butylpyridine (Sigma-Aldrich, St. Louis, MO, USA) and 17.5 μL of a lithium bis(trifluoromethanesulfonyl)imide solution (Li-TFSI; Sigma-Aldrich, St. Louis, MO, USA) in acetonitrile (Sigma-Aldrich, St. Louis, MO, USA). Au top electrodes with a thickness of 80 nm were deposited by thermal evaporation under a base pressure of approximately 2 × 10^−6^ Torr. The Au electrode geometry was kept identical for all devices to ensure consistent electrical comparison.

These layers were deposited after the perovskite patterning process, while the patterned and unpatterned regions remained part of the same continuous perovskite layer.

### 2.6. Electrical and Optoelectronic Characterization

The current–voltage (I–V) characteristics of the photodetectors were measured using a source-measure unit (Keithley 2400, Cleveland, OH, USA) under dark and UV-illumination conditions. The photocurrent measurements were performed under 254 nm UV-C illumination using a UVGL-58 lamp (Analytik Jena, Jena, Germany) positioned approximately 10 cm from the device surface, with an optical power density of approximately 1.2 mW cm^−2^ based on the manufacturer’s specifications. UV-C light was applied without the patterning mask to the central device region, excluding the electrode regions, corresponding to an illuminated area of approximately 2.0 cm^2^. The measurement duration was not systematically recorded and therefore could not be included as a controlled experimental parameter. Temporal response characteristics, including response speed, were not evaluated in the present study. For the patterning-time-dependent electrical characterization, five independently fabricated devices were measured for each UV-C patterning condition (*n* = 5) at a bias voltage of 1 V. Photocurrent and dark-current values are reported as mean ± standard deviation to evaluate device-to-device variation. Responsivity, EQE, and specific detectivity (D*) were calculated from the corresponding electrical characteristics obtained from these devices.

The responsivity (R), external quantum efficiency (EQE), and specific detectivity (D*) were calculated using the following equations:

(1) $$R\;=\;\frac{I_{\mathrm{photo}}-I_{\mathrm{dark}}}{P_{\mathrm{in}}}$$

(2) $$\mathrm{EQE}=\frac{\mathrm{Rhc}}{\mathrm{q\lambda}}$$

(3) $$D*=\frac{R\sqrt{A}}{\sqrt{2qI_{\mathrm{dark}}}}$$

where *I_photo_* and *I_dark_* are the photocurrent and dark current, respectively, *P_in_* is the incident optical power, *h* is Planck’s constant, *c* is the speed of light, *q* is the elementary charge, *λ* is the incident wavelength, and *A* is the active device area. The specific detectivity was estimated under a shot-noise-limited assumption, in which the dominant noise contribution was assumed to originate from the dark current [25].

## 3. Results

### 3.1. Selective Surface Thinning Induced by Patterning

The overall device architecture and fabrication process are illustrated in Figure 1, and the patterning process is illustrated in Figure 2. Based on this device configuration, patterning was performed to selectively modify the surface region of the mixed-cation perovskite films.

**Figure 3:**
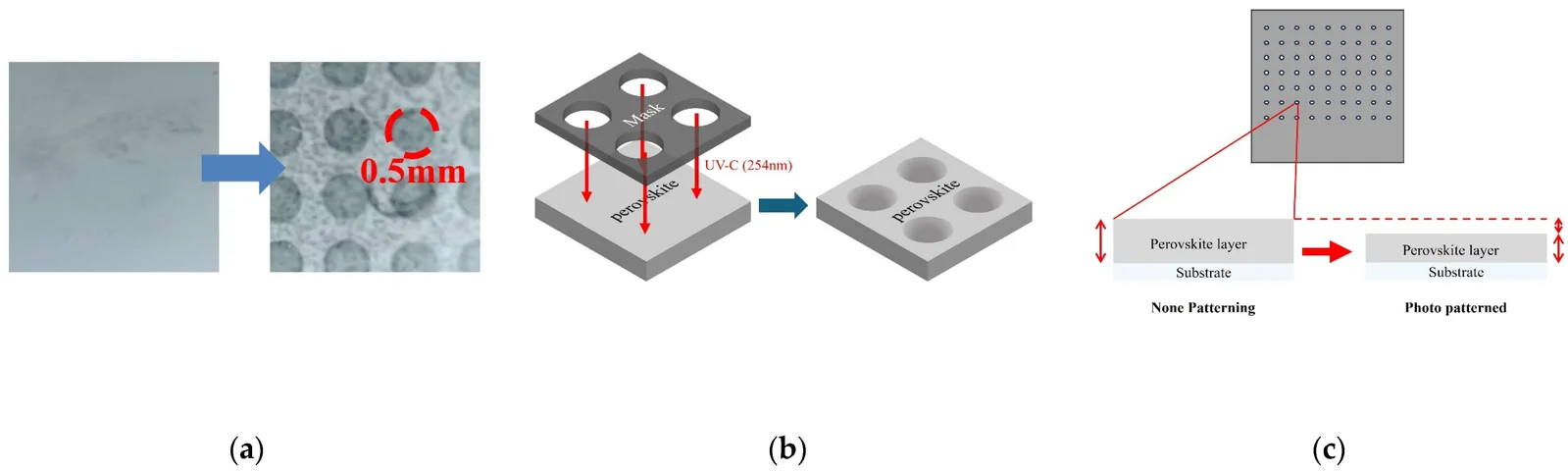
Formation of photopatterned perovskite films by patterning. (**a**) Optical microscopy images of a mixed-cation perovskite film before and after patterning, showing well-defined circular patterns with a diameter of 0.5 mm; (**b**) schematic highlighting localized surface modification induced by UV-C irradiation through a shadow mask; (**c**) schematic of selective near-surface thinning induced by UV-light-assisted patterning through a shadow mask. The red dashed circle indicates the 0.5 mm pattern diameter, the red arrows indicate the direction of UV-C irradiation, and the red lines and arrows illustrate the change in perovskite film thickness before and after patterning.

As shown in Figure 3, irradiation of the perovskite layer through a shadow mask resulted in the formation of well-defined circular patterns on the film surface. The patterned features exhibited clear spatial contrast relative to the surrounding unexposed areas, demonstrating spatially selective modification of the UV-C-exposed regions.

Both regions remained physically continuous within the perovskite film; however, their individual contributions to the measured electrical response were not spatially resolved in the present study.

Importantly, no macroscopic film damage or delamination was observed under the investigated patterning conditions.

#### 3.1.1. Morphological Evidence of Surface Thinning

The morphological impact of patterning on mixed-cation perovskite films was investigated using cross-sectional analysis. The perovskite films maintained continuous coverage without visible delamination or collapse of the underlying layers after UV-C patterning.

**Figure 4:**
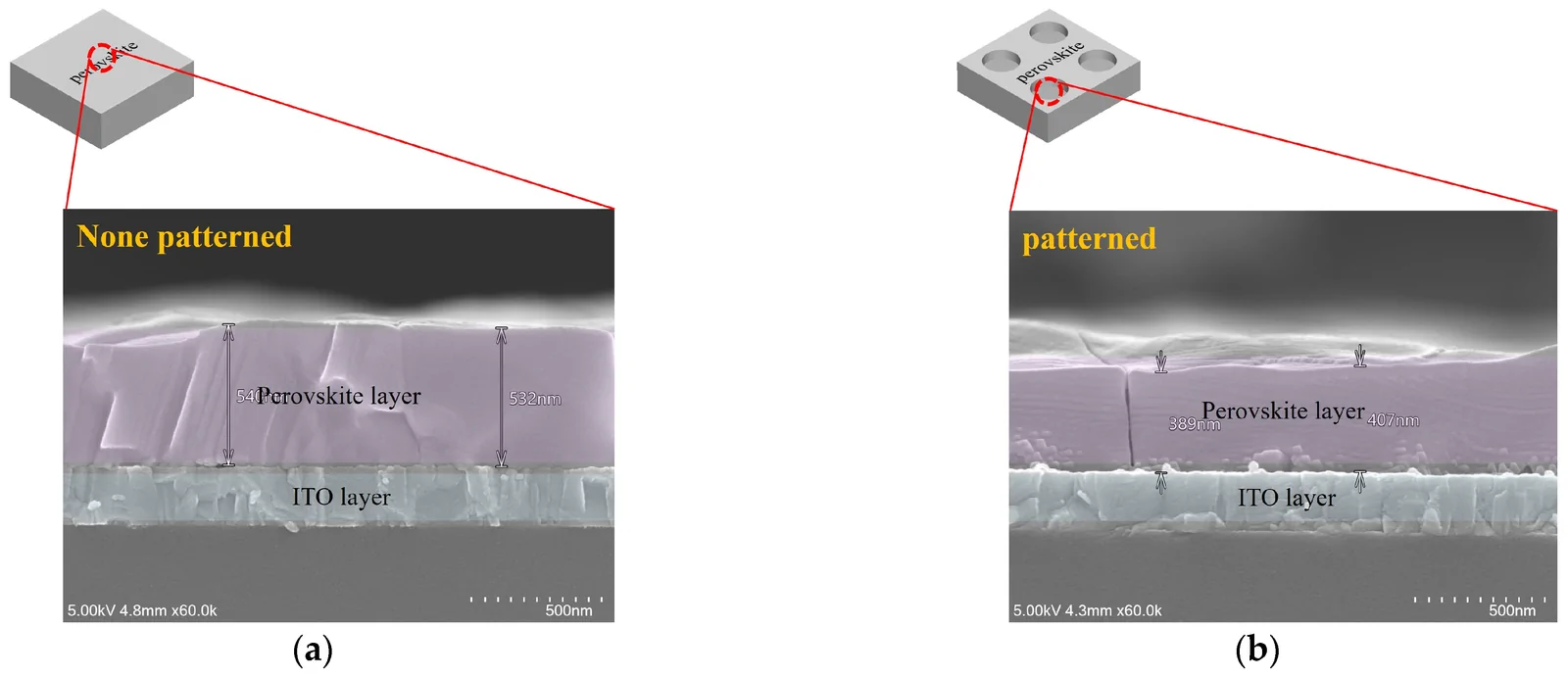
Cross-sectional SEM analysis of selective surface thinning in patterned perovskite films. (**a**) Cross-sectional SEM image of a non-patterned mixed-cation perovskite film, showing a uniform film thickness of approximately 530–540 nm; (**b**) cross-sectional SEM image of a photopatterned perovskite film after 7 min of UV-C patterning, revealing a reduced film thickness of approximately 390–410 nm with preservation of the underlying layer structure.

As shown in Figure 4a,b, the non-patterned perovskite film exhibited a uniform thickness of approximately 530–540 nm, whereas the photopatterned film exposed to UV-C light for 7 min showed a reduced thickness of approximately 390–410 nm. The thickness reduction was observed in the perovskite layer, while the underlying layers remained intact. The perovskite-layer thickness was measured at three different positions within each cross-sectional SEM image, and the reported values represent the approximate thickness ranges obtained from these measurements rather than a value measured at a single representative position.

To further examine the thickness modulation under different patterning conditions, alpha-step profilometry measurements were performed at three different positions on each sample (*n* = 3 measurement positions). The remaining perovskite film thicknesses were 479.7 ± 11.2, 420.3 ± 4.0, and 350.7 ± 4.2 nm after UV-C patterning for 3, 7, and 10 min, respectively.

#### 3.1.2. Thickness Modulation Under Different Patterning Conditions

**Figure 5:**
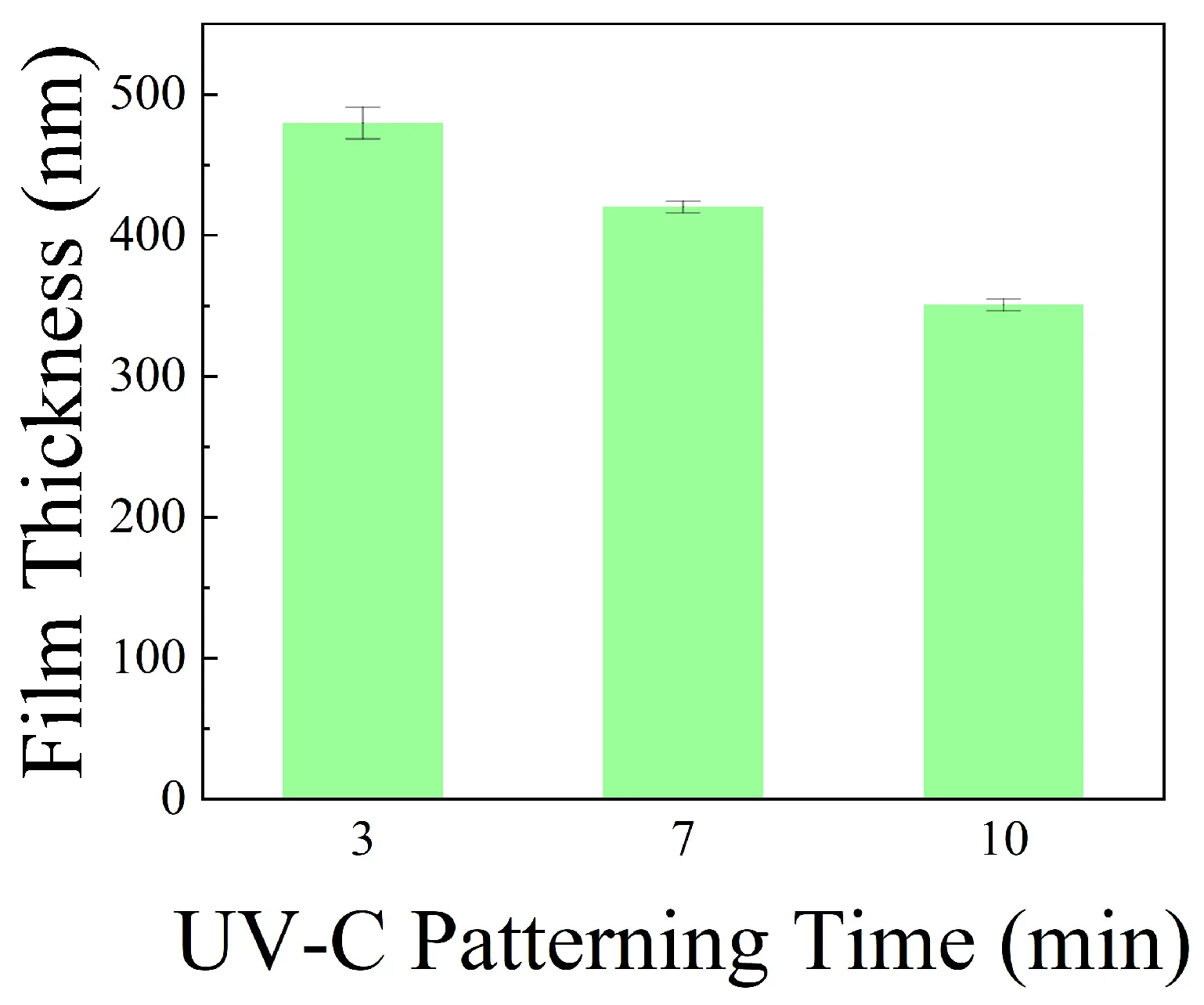
Patterning-time-dependent thickness modulation of the perovskite film measured by alpha-step profilometry. The remaining film thickness was measured at three different positions on each sample after UV-C patterning for 3, 7, and 10 min. Data are presented as mean ± standard deviation (*n* = 3 measurement positions).

To examine the patterning-time-dependent trend in surface thinning, Figure 5 presents the remaining perovskite film thickness measured by alpha-step profilometry as a function of UV-C patterning time. The progressive decrease in film thickness with increasing patterning time indicates a patterning-time-dependent thinning trend under the investigated conditions.

The remaining perovskite film thickness decreased from 479.7 ± 11.2 nm at 3 min to 420.3 ± 4.0 nm at 7 min and 350.7 ± 4.2 nm at 10 min, confirming a progressive patterning-dependent thinning trend.

Based on these results, a patterning time of 7 min was selected for subsequent device fabrication and optoelectronic characterization.

### 3.2. Crystallographic and Optical Characteristics After Perovskite Patterning

#### 3.2.1. X-Ray Diffraction Analysis

**Figure 6:**
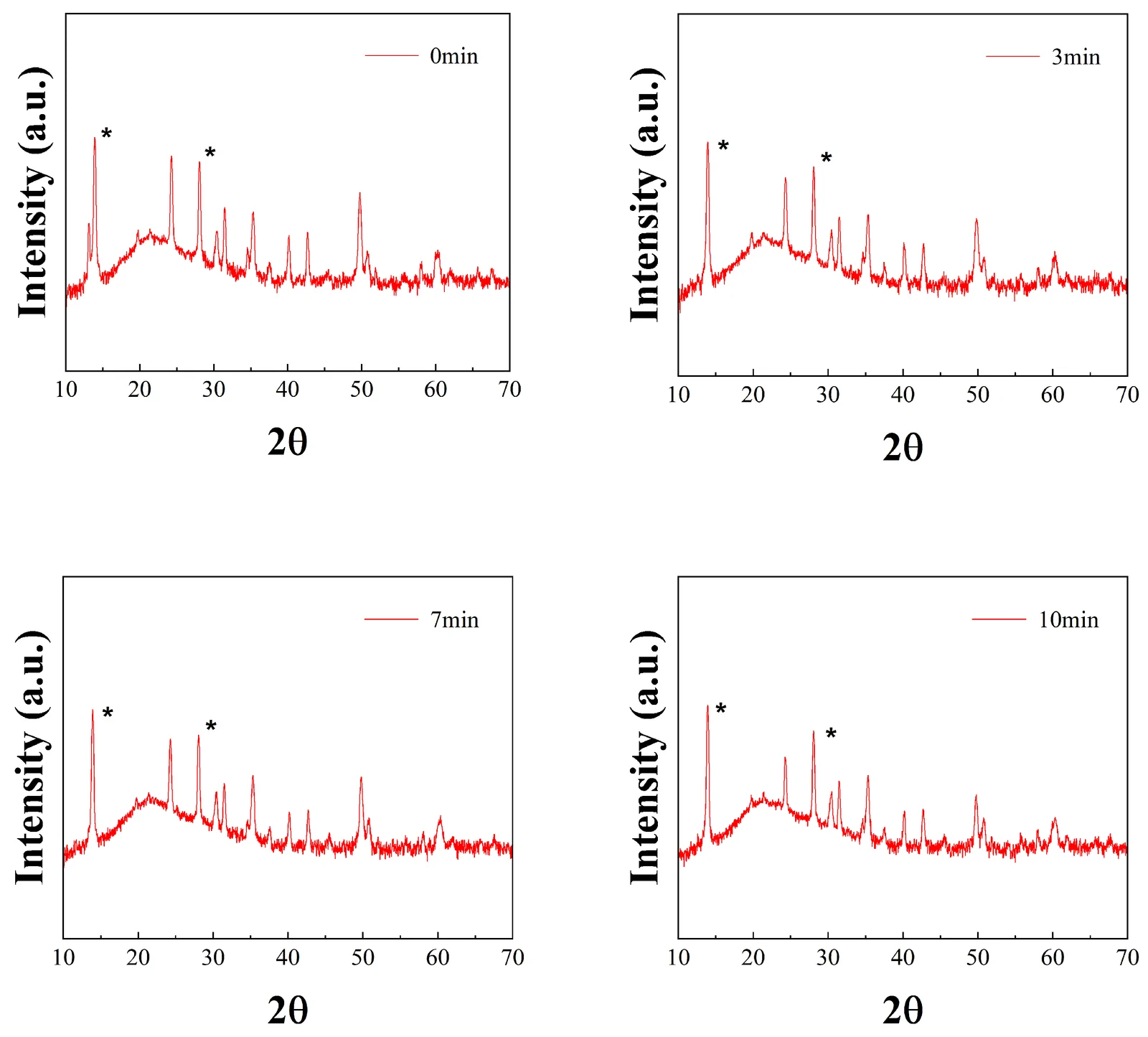
X-ray diffraction (XRD) analysis of perovskite films before and after patterning under varying UV-C patterning times. The diffraction peaks marked with asterisks (*) indicate the characteristic peaks of the perovskite phase.

To evaluate the structural characteristics of the perovskite films after patterning, X-ray diffraction (XRD) analysis was performed on the pristine and photopatterned samples. Figure 6 compares the XRD patterns of the perovskite films before and after UV-C patterning.

The asterisk-marked diffraction peaks located at approximately 14.0°, 20.0°, 24.5°, 28.2°, and 31.7°, corresponding to the (110), (112), (202), (220), and (310) crystal planes of the perovskite structure, respectively, were preserved after UV-C patterning. No diffraction peak was observed at approximately 12.6°, corresponding to the (001) plane of PbI_2_ [26,27].

No noticeable peak broadening, peak shift, or emergence of secondary phases was observed. In addition, no significant variation in peak positions or intensities was observed across the investigated UV-C patterning times.

#### 3.2.2. UV–Vis Absorption Analysis

**Figure 7:**
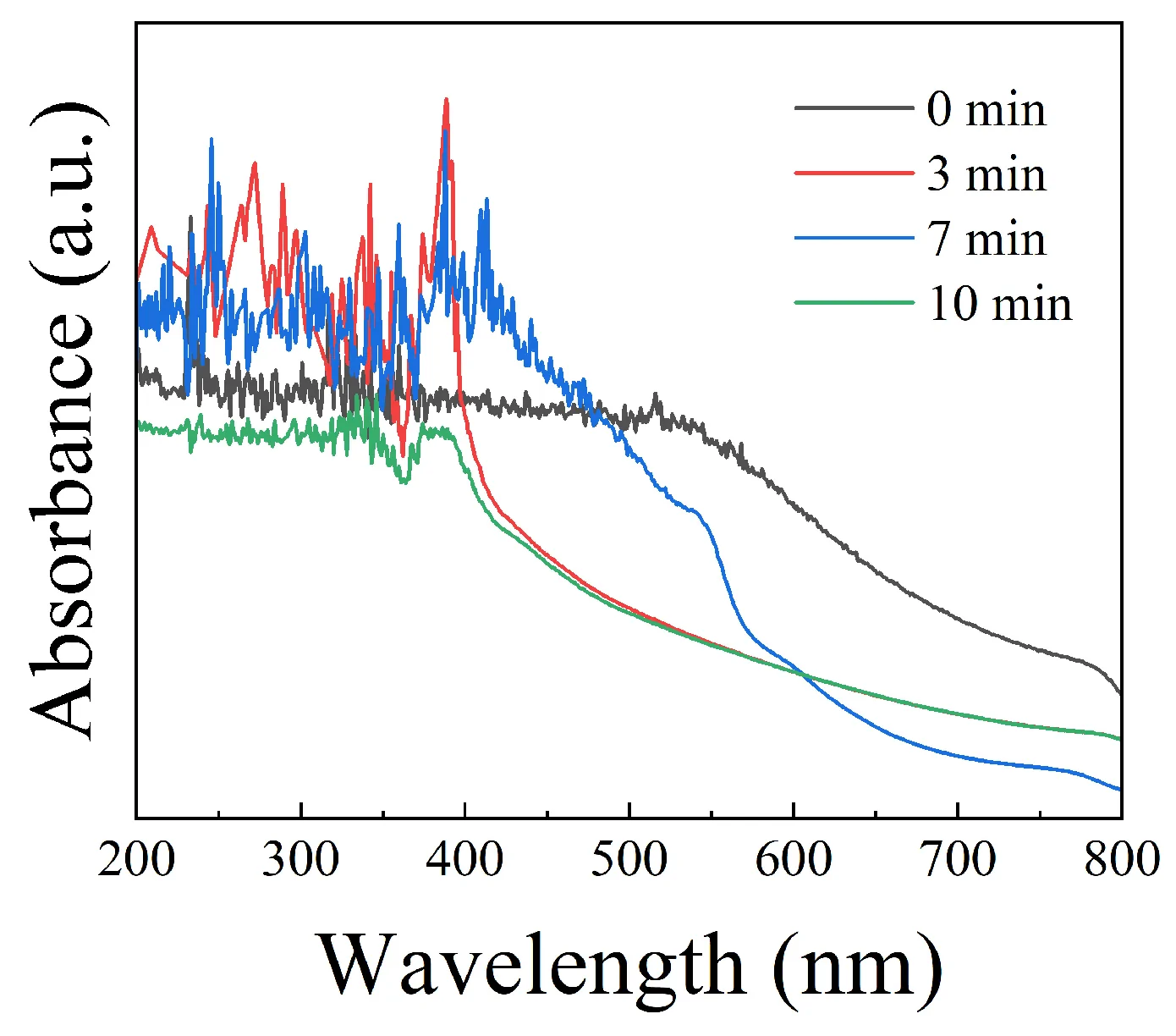
UV–vis absorption spectra of perovskite films subjected to different UV-C patterning times (0, 3, 7, and 10 min).

The optical absorption properties of the perovskite films were examined before and after patterning. Figure 7 shows the UV–vis absorption spectra of the perovskite films subjected to patterning times of 0, 3, 7, and 10 min.

The absorption onset and overall spectral profile remained nearly unchanged after UV-C patterning. A slight reduction in the absolute absorbance intensity was observed for the photopatterned films, while no pronounced spectral distortion or band-edge shift was observed.

### 3.3. Photoresponse of Patterned Mixed-Cation Perovskite Devices

#### 3.3.1. Patterning-Time-Dependent Electrical Characteristics

**Figure 8:**
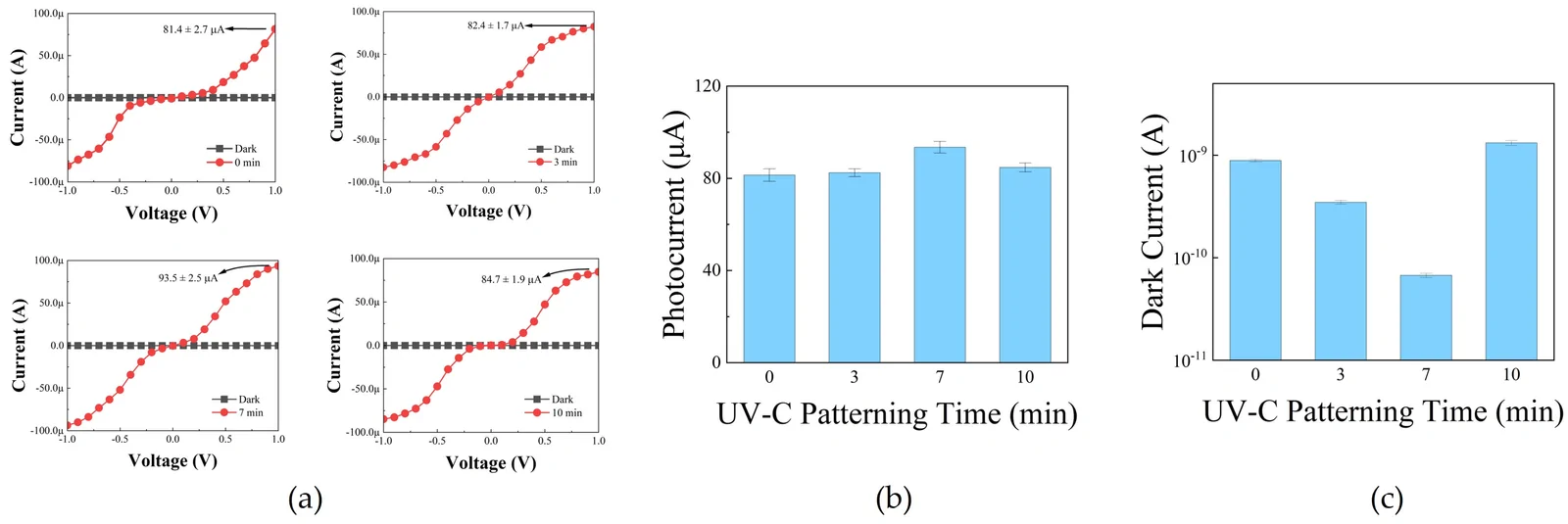
Electrical characteristics of mixed-cation perovskite photodetectors as a function of UV-C patterning time. (**a**) Representative current–voltage (I−V) characteristics measured under dark and UV-illumination conditions for patterning times of 0, 3, 7, and 10 min. (**b**) Photocurrent and (**c**) dark current as a function of UV-C patterning time. The values in (**b**) and (**c**) were obtained at a bias voltage of 1 V and are presented as the mean ± standard deviation from five independently fabricated devices for each condition (*n* = 5). The dark-current values in (**c**) are plotted on a logarithmic scale.

The effect of patterning time on the electrical characteristics of the perovskite photodetectors was investigated by analyzing the current–voltage (I–V) behavior under dark and UV-illumination conditions. Figure 8a shows the representative I–V characteristics of devices subjected to UV-C patterning times of 0, 3, 7, and 10 min, while Figure 8b,c summarize the corresponding photocurrent and dark-current statistics, respectively.

In the dark state, the photopatterned devices exhibited a reduction in dark current as the UV-C patterning time increased up to 7 min. Among the investigated conditions, the device photopatterned for 7 min exhibited the lowest dark current. Further patterning to 10 min did not produce additional appreciable suppression of the dark current.

Under UV illumination, the photopatterned devices exhibited higher photocurrent than the non-patterned reference. The photocurrent increased with patterning time up to 7 min, whereas further patterning to 10 min produced no additional improvement.

The combined reduction in dark current and enhancement in photocurrent resulted in an increased photo-to-dark-current ratio under the optimized patterning condition.

#### 3.3.2. Pattern-Diameter-Dependent Electrical Characteristics

After identifying 7 min as the optimized UV-C patterning time, the effect of pattern diameter was evaluated using circular patterns of 0.3, 0.5, and 1.0 mm. For each pattern diameter, five independently fabricated devices were measured (*n* = 5). The mean photocurrent values were approximately 92.8, 93.5, and 88.6 μA for pattern diameters of 0.3, 0.5, and 1.0 mm, respectively. The 0.3 mm patterns showed less clearly defined features because of their small dimensions, whereas the 1.0 mm patterns exhibited signs of excessive surface modification associated with the larger exposed area. In contrast, the 0.5 mm condition provided a more uniform patterned morphology and the highest photocurrent among the investigated diameters. Therefore, 0.5 mm was selected as the optimal pattern diameter for subsequent device characterization.

**Figure 9:**
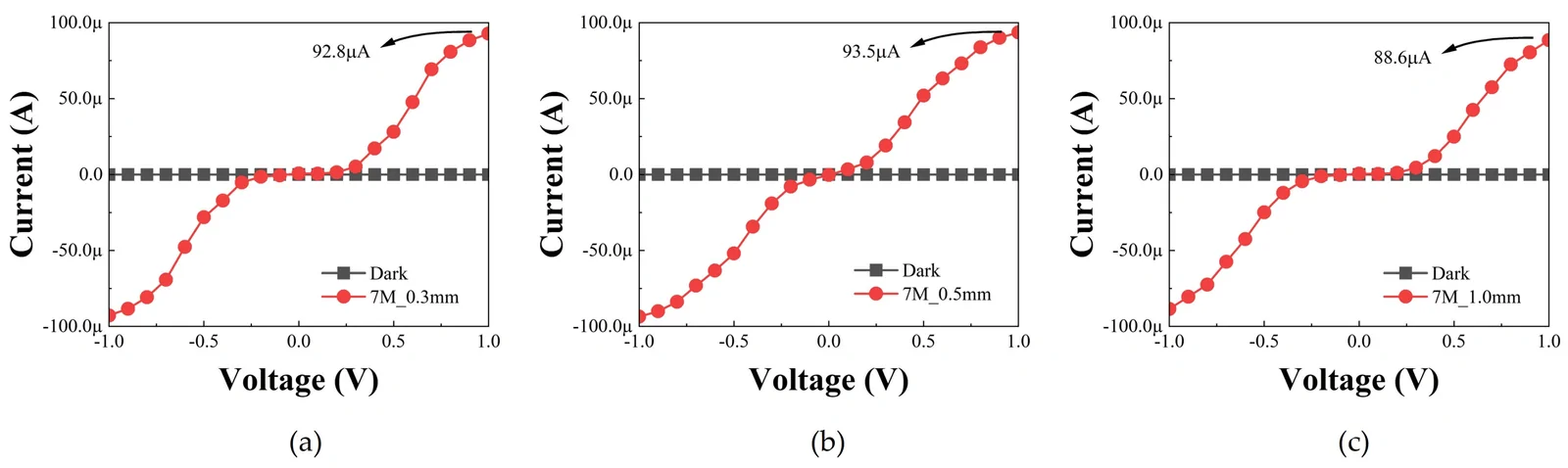
Representative current–voltage characteristics of perovskite photodetectors patterned for 7 min with different circular pattern diameters: (**a**) 0.3 mm, (**b**) 0.5 mm, and (**c**) 1.0 mm. Five independently fabricated devices were evaluated for each pattern diameter (*n* = 5).

Figure 9 shows the representative current–voltage characteristics of the perovskite photodetectors patterned for 7 min with circular pattern diameters of 0.3, 0.5, and 1.0 mm.

#### 3.3.3. Optoelectronic Performance Metrics

To quantitatively evaluate the optoelectronic performance of the perovskite photodetectors, responsivity (R), external quantum efficiency (EQE), and specific detectivity (D*) were analyzed as a function of patterning time.

**Figure 10:**
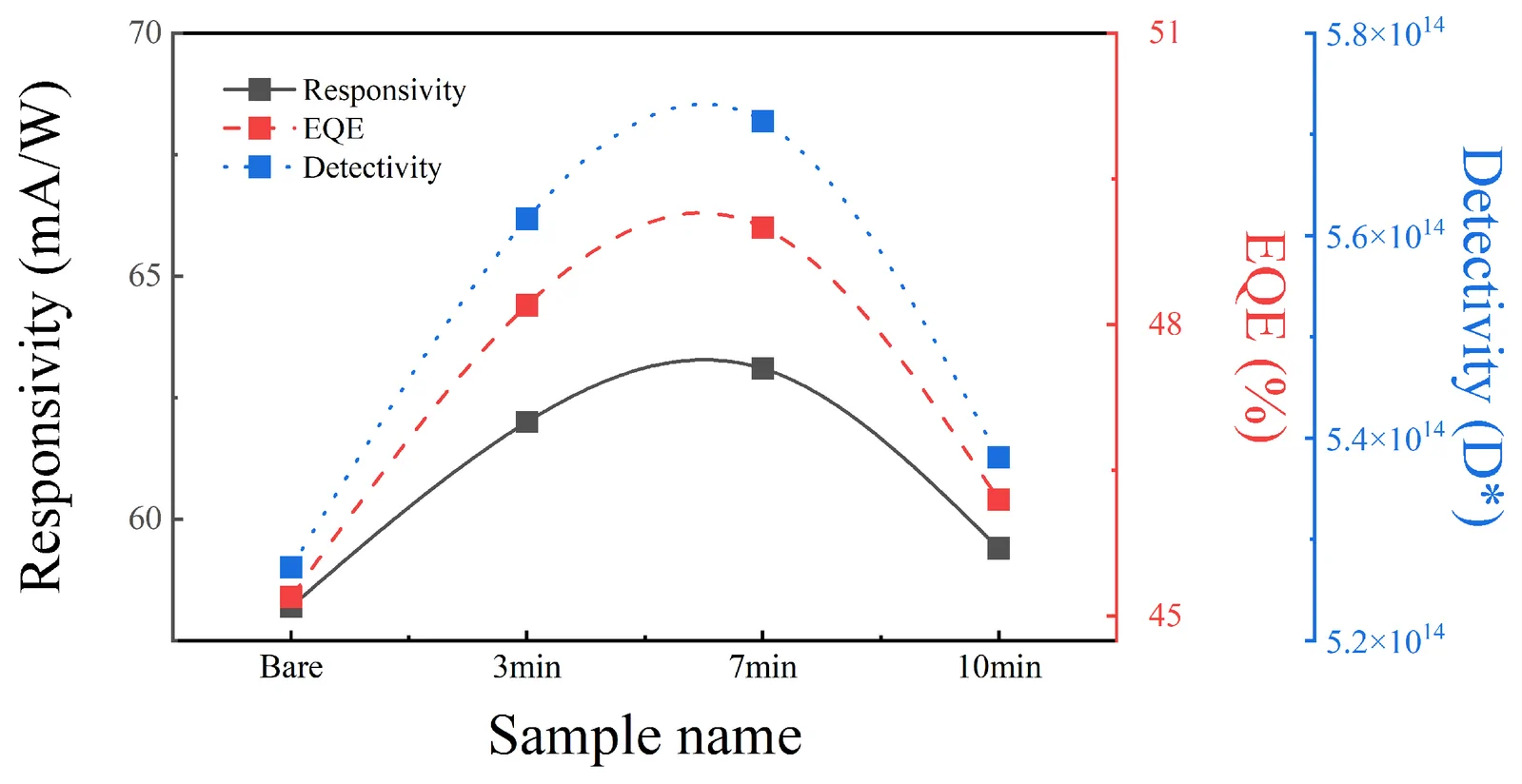
Variation in responsivity, external quantum efficiency (EQE), and specific detectivity (D*) of mixed-cation perovskite photodetectors as a function of UV-C patterning time (0, 3, 7, and 10 min). The reported parameters were calculated based on electrical measurements obtained from five independently fabricated devices for each patterning condition (*n* = 5).

Figure 10 summarizes the variations in these parameters for devices subjected to UV-C patterning times of 0, 3, 7, and 10 min.

As the patterning time increased from 0 to 7 min, responsivity, EQE, and specific detectivity increased, followed by a decrease at 10 min. Among the investigated patterning conditions, the 7 min condition exhibited the highest mean electrical and optoelectronic performance. Therefore, 7 min was selected as the optimized processing condition within the range of patterning times investigated in this study.

## 4. Discussion

### 4.1. UV-C-Induced Surface Modification of Perovskite Films

The optical microscopy, cross-sectional SEM, and profilometry results collectively demonstrate that ultraviolet light irradiation through a shadow mask induces localized thickness modulation in the exposed regions of the perovskite film. The decrease in the remaining perovskite film thickness from 479.7 ± 11.2 nm at 3 min to 420.3 ± 4.0 nm at 7 min and 350.7 ± 4.2 nm at 10 min demonstrates an exposure-dependent thinning trend with increasing UV-C irradiation time.

The progressive decrease in the remaining film thickness with increasing irradiation time indicates a time-dependent surface-modification behavior under UV-C exposure. Light-based surface modification of perovskite films has also been demonstrated using controlled UV-laser processing, highlighting the possibility of modifying perovskite surface properties through carefully controlled irradiation [28].

Importantly, the morphological results should be considered together with the XRD measurements. Despite the substantial reduction in film thickness after patterning, the characteristic perovskite diffraction peaks remained largely unchanged, and no PbI_2_-related diffraction peak at approximately 12.6° was observed [26,27]. These observations indicate that the patterning conditions used in this study predominantly modify the near-surface region without producing detectable bulk crystallographic degradation.

The UV–vis measurements further support this interpretation. The absence of a pronounced shift in the absorption onset or major distortion of the spectral profile indicates that the overall optical characteristics of the remaining perovskite layer are preserved. The slight reduction in absolute absorbance after patterning can be associated with the reduced effective film thickness resulting from surface thinning.

Taken together, the morphological, structural, and optical analyses indicate that controlled UV-C irradiation provides a processing window in which surface thickness can be modulated while the principal crystallographic and optical characteristics remain largely unchanged under the investigated conditions.

### 4.2. Effect of Surface Patterning on Photodetector Performance

The electrical measurements show that controlled perovskite patterning simultaneously suppresses the dark current and enhances the photocurrent up to a patterning time of 7 min. This behavior suggests that the surface modification influences both the electrical leakage pathways and photocarrier transport within the device.

The reduction in dark current may be associated with modification of the near-surface region, where defect-related leakage and nonradiative recombination can strongly influence photodetector characteristics [19,20]. Previous studies have also shown that controlled light-induced surface modification of perovskite films can alter their surface characteristics [28], providing a possible basis for interpreting the electrical trends observed in the present study. At the same time, controlled thinning of the perovskite layer may alter the interaction between incident UV light and the active layer and reduce the effective transport distance of photogenerated carriers.

The concurrent increases in responsivity, EQE, and specific detectivity with patterning time up to 7 min are consistent with the observed enhancement in photocurrent and reduction in dark current. In particular, the increase in detectivity reflects the combined contribution of enhanced photoresponse and suppressed dark-current characteristics.

**Figure 11:**
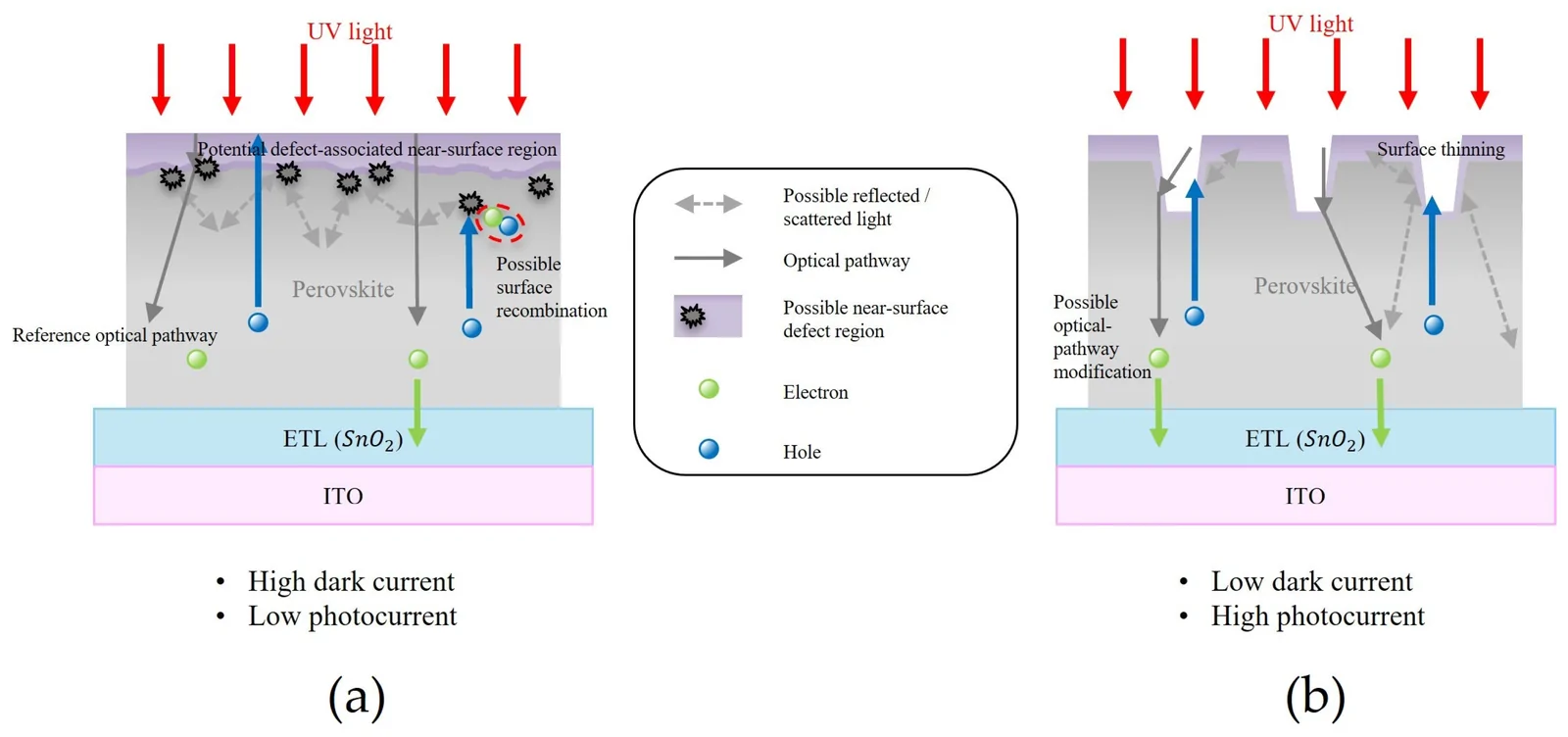
Schematic illustration of possible light-pathway and carrier-transport modifications in perovskite UV photodetectors: (**a**) non-patterned device; (**b**) patterned device.

To illustrate the proposed relationship between surface modification and device performance, the light-pathway and carrier-transport mechanism is schematically presented in Figure 11.

As illustrated in Figure 11, in the non-patterned device, incident UV light is absorbed within the perovskite layer, including the near-surface region where defect-assisted recombination may occur. Following controlled patterning, thinning of the exposed surface region may alter the optical pathway within the perovskite layer. Because optical simulations and direct measurements of light penetration or scattering were not performed in the present study, these effects are considered possible consequences of the modified morphology rather than experimentally confirmed mechanisms. This modification may contribute to enhanced photocarrier generation and extraction.

Controlled thinning may also reduce the effective transport distance between the photocarrier-generation region and the charge-collecting interface. The combined modification of the optical pathway and carrier-transport conditions provides a possible explanation for the simultaneous enhancement in photocurrent and suppression of dark current observed in the patterned devices.

Previous studies have shown that structural and morphological modification can influence light trapping, optical pathways, and photon utilization in perovskite and related optoelectronic structures [29,30,31,32,33,34]. In addition, patterned or nanostructured perovskite architectures have been reported to exhibit modified optical behavior and enhanced optoelectronic performance [35,36,37]. These previous findings provide supporting context for the possible optical effects proposed here, although they do not directly confirm the mechanism in the present devices.

### 4.3. Optimization of UV-C Patterning Time

Although the degree of surface thinning increased continuously with UV-C patterning time, device performance did not increase monotonically. Instead, the responsivity, EQE, and specific detectivity reached their maximum values after 7 min of patterning and decreased slightly when the patterning time was extended to 10 min.

This difference between the thickness-modulation trend and the device-performance trend indicates that maximizing surface thinning alone does not maximize photodetector performance. Rather, an appropriate balance between surface modification and preservation of the perovskite layer is required.

The slight degradation in device performance after 10 min of patterning may be associated with excessive surface modification or increased recombination losses under prolonged ultraviolet light irradiation. However, this interpretation remains a possible hypothesis because direct characterization of defect states or recombination dynamics was not performed in the present study.

Accordingly, among the investigated conditions, the 7 min patterning condition exhibited the highest mean device performance and was therefore selected as the optimized processing condition within the investigated patterning-time range.

In addition to the patterning-time optimization, the pattern diameter affected both the patterned morphology and the photocurrent response. The 0.5 mm condition exhibited the highest average photocurrent among the investigated diameters while maintaining a more uniform patterned morphology than the 0.3 and 1.0 mm conditions, supporting its selection as the optimal pattern size.

To further evaluate the performance of the optimized device, the key photodetection parameters were compared with those of representative perovskite-based UV-C photodetectors reported previously, as summarized in Table 1 [38,39,40]. The optimized patterned device exhibited a responsivity of 63.1 mA W^−1^, an EQE of 49.0%, and a specific detectivity of 5.713 × 10^14^ Jones at 254 nm.

Although the optimized patterning conditions improved the photodetection performance, the long-term operational stability of the patterned devices was not evaluated in the present study. Future work should therefore investigate device stability under repeated or prolonged UV-C exposure to determine whether the patterning process affects long-term performance retention.

## 5. Conclusions

In this study, controlled perovskite patterning using ultraviolet light irradiation was investigated as a surface-engineering strategy for enhancing the performance of perovskite UV photodetectors. UV-C irradiation using a shadow mask produced localized surface modification and controllable thinning of the mixed-cation perovskite layer.

Cross-sectional SEM and profilometry measurements showed that the perovskite film thickness progressively decreased with increasing ultraviolet light exposure time, with remaining thicknesses of 479.7 ± 11.2, 420.3 ± 4.0, and 350.7 ± 4.2 nm after patterning for 3, 7, and 10 min, respectively. Despite this thickness modulation, XRD and UV–vis analyses showed that the principal crystallographic and optical characteristics of the perovskite films were largely preserved under the investigated conditions.

Electrical and optoelectronic measurements demonstrated that controlled patterning reduced the dark current and enhanced the photocurrent. Responsivity, EQE, and specific detectivity reached their highest values at a patterning time of 7 min, whereas further patterning to 10 min resulted in a slight decrease in device performance. These results indicate that an appropriate balance between surface modification and material integrity is important for maximizing device performance.

Overall, this study demonstrates that controlled ultraviolet light irradiation can be utilized as a surface-patterning approach for perovskite photodetectors rather than being treated solely as a degradation source. The proposed approach provides a simple strategy for modifying the perovskite surface and improving UV photodetector performance.

## 6. Patents

A Korean patent application related to this work has been filed: Choi, H.W.; Kim, J.; Kim, S. Perovskite-based ultraviolet photodetector and its manufacturing method, Korean Patent Application No. 10-2026-0051749, filed on 23 March 2026.

## Figures and Tables

**Table 1:** Comparison of key photodetection parameters of representative perovskite-based UV-C photodetectors.

Perovskite-Based UV-C Photodetector	Operating Wavelength (nm)	Responsivity (mA W^−1^)	EQE (%)	Specific Detectivity (Jones)	Rise/Fall Time	Ref.
FA_X_MA_1-X_PbI_3_ mixed-cation PD	254	50.8	53.0	4.47 × 10^13^	223/272 ms	[38]
CsPbI_2_Br solar-blind PD	255	56.2	N.R.	2.86 × 10^13^	N.R.	[39]
CsPbBr_3_–Cs_4_PbBr_6_/hybrid perovskite PD	254	49.4	N.R.	1.2 × 10^12^	7.8/33.6 μs	[40]
This work (7 min, 0.5 mm)	254	63.1	49.0	5.713 × 10^14^	Not measured	This work

## Data Availability

The original contributions presented in this study are included in the article. Further inquiries can be directed to the corresponding authors.
